# Predicting individual responses to the electroconvulsive therapy with hippocampal subfield volumes in major depression disorder

**DOI:** 10.1038/s41598-018-23685-9

**Published:** 2018-04-03

**Authors:** Bo Cao, Qinghua Luo, Yixiao Fu, Lian Du, Tian Qiu, Xiangying Yang, Xiaolu Chen, Qibin Chen, Jair C. Soares, Raymond Y. Cho, Xiang Yang Zhang, Haitang Qiu

**Affiliations:** 1 Mental Health Center, The First Affiliated Hospital of Chongqing Medical University, Chongqing, P. R. China; 2 Department of Psychiatry and Behavioral Sciences, McGovern Medical School, The University of Texas Health Science Center at Houston, Houston, United States; 3 Department of Anesthesiology, The First Affiliated Hospital of Chongqing Medical University, Chongqing, P. R. China

**Keywords:** Predictive markers, Depression

## Abstract

Electroconvulsive therapy (ECT) is one of the most effective treatments for major depression disorder (MDD). ECT can induce neurogenesis and synaptogenesis in hippocampus, which contains distinct subfields, e.g., the cornu ammonis (CA) subfields, a granule cell layer (GCL), a molecular layer (ML), and the subiculum. It is unclear which subfields are affected by ECT and whether we predict the future treatment response to ECT by using volumetric information of hippocampal subfields at baseline? In this study, 24 patients with severe MDD received the ECT and their structural brain images were acquired with magnetic resonance imaging before and after ECT. A state-of-the-art hippocampal segmentation algorithm from Freesurfer 6.0 was used. We found that ECT induced volume increases in CA subfields, GCL, ML and subiculum. We applied a machine learning algorithm to the hippocampal subfield volumes at baseline and were able to predict the change in depressive symptoms (r = 0.81; within remitters, r = 0.93). Receiver operating characteristic analysis also showed robust prediction of remission with an area under the curve of 0.90. Our findings provide evidence for particular hippocampal subfields having specific roles in the response to ECT. We also provide an analytic approach for generating predictions about clinical outcomes for ECT in MDD.

## Introduction

Major depression disorder (MDD) is one of the most prevalent and disabling mental disorders across the world^1,2^. Although several pharmaceutical treatment options are available to patients with MDD, electroconvulsive therapy (ECT) is considered as the most effective treatment for severe MDD^3,4^. The traditional ECT was modified to be administered during anesthesia (modified ECT), in order to reduce patients’ discomfort during the procedure. However, due to the historical stigma, associated cognitive impairments, and financial burdens, ECT remains a challenging therapeutic option to consider for patients and clinicians.

Recent research employing machine learning and magnetic resonance imaging (MRI) may help the patients and psychiatrists to achieve more informed decisions regarding ECT as a therapeutic option^5,6^. These studies used machine learning algorithms to identify the patients that were most likely to benefit from ECT at an individual level. The algorithms also helped to discover the biomarkers in the brain that were predictive of ECT treatment response.

One of the biomarkers that have been associated with ECT is hippocampal volume. The hippocampus is a site of active neurogenesis and neuroplasticity and ECT may induce neurogenesis, synaptogenesis, and glial proliferation^7–9^. Hippocampal volumes were reported to increase after ECT^10–12^. However, hippocampus is not a uniform brain structure and contains several subfields with distinct anatomical and functional features, such as the cornu ammonis (CA) subfields CA1-4, the dentate gyrus that contains a granule cell layer (GCL) and a molecular layer (ML), and the adjacent subiculum and presubiculum^13^. Previous findings indicated that different mental disorders might involve different mechanisms within hippocampus during the progression of the illness^14–20^. However, subfields that are involved in the cortical connections, mediating connections within the hippocampus and neurogenesis were considered sensitive to the mood disorder episodes, especially the manic episode, such as CA2/3, CA4, GCL, ML and subiculum^15^. These subfields might in turn be responsive to the seizure-induced neuronal changes due to ECT. A previous study showed that volumetric increase in bilateral CA2/3 and right hippocampal subfields might be specifically associated with ECT^21^. A recent hippocampal segmentation method could provide more accurate estimation of hippocampal subfield volumes than prior methods^14,21–23^. However, it remains unclear which hippocampal subfields were reliably affected by ECT and whether subfield volumes at baseline could be predictive of ECT treatment response.

In the current study, we aimed to investigate the volumetric changes of hippocampal subfields in patients with MDD who received ECT using a state-of-the-art hippocampal segmentation approach. We hypothesized that ECT would induce volume increases in CA2/3, CA4, GCL, ML and subiculum. We also aimed to predict the treatment response to ECT by using machine learning and hippocampal subfield volumes at baseline, the success of which will help us make personalized clinical suggestions for patients who are suffering from MDD and may potentially benefit from ECT.

## Results

### Demographics

Twenty-four severe MDD inpatients (14 females and 10 males, aged 31.3 ± 10.8) were recruited. Fifteen healthy subjects (10 females and 5 males, aged 33.1 ± 10.0) were recruited as healthy controls (HC). There was no significant difference in age or gender between patients with MDD and HC (p > 0.05; Table 1). HC had significantly higher education compared to patients (F_1,37_ = 17.937, p < 0.0001). Patients with MDD had an average of HAM-D total scores as high as 31.3, which was significantly different from that of HC (F_1,36_ = 544.505, p < 0.0001; one HC had missing HAM-D scores).

**Table 1:** Demographic information of the subjects.

	HC (n = 15)	MDD (n = 24)	F/X^2^	P value
Age (years)	33.1 ± 10.0	31.3 ± 10.8	0.251	0.619
Gender			0.271	0.603
Male	33.3% (5)	41.7% (10)		
Female	66.7% (10)	58.3% (14)		
Education (years)	15.3 ± 3.4	11.0 ± 2.8	17.937	<0.001
HAMD	2.2 ± 1.3	31.3 ± 4.5	544.505	<0.001

### Efficacy of ECT

The average reduction of HAM-D scores after ECT was significant (22.75 ± 7.18; t = 15.517; p < 0.001). Twenty-two MDD patients (91.7%) showed significant improvement (more than 50% decrease of HAM-D scores) after receiving ECT. Twelve patients (50%) were considered to be in remission, as their HAM-D scores were equal or less than 7. Age, gender, education and HAM-D were not different between the remitters and non-remitters of ECT (all p > 0.05).

### Results of group-level analyses at baseline

**Figure 1:**
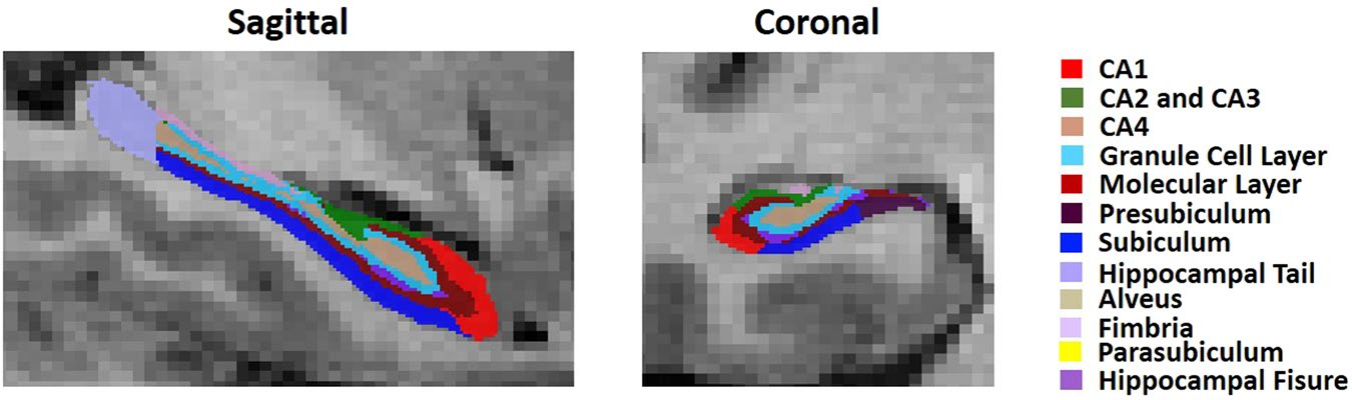
Hippocampal subfield segmentation sample of a patient with major depression disorder. CA, cornu ammonis.

**Figure 2:**
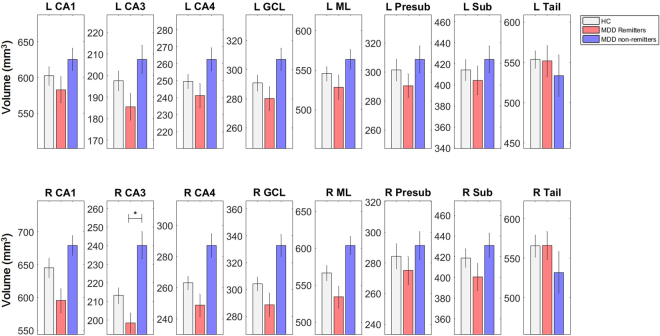
Baseline hippocampal subfield volumes of healthy controls, remitters and non-remitters of ECT. MDD, major depressive disorder; CA, cornu ammonis; GCL, granule cell layer; ML, molecular layer; Presub, presubiculum; Sub, subiculum and Tail, hippocampal tail. L, left; R, right.

The hippocampal subfields were labeled with a novel segmentation algorithm (Fig. 1)^22^. No volume difference was observed in any of the hippocampal subfields between MDD and HC at baseline. Post-hoc analysis found significantly lower volumes in right CA1, CA3, CA4, GCL and ML of the remitters than the non-remitters, while only right CA3 survived the Bonferroni correction (Fig. 2).

### Results of group-level analyses in MDD before and after ECT

**Figure 3:**
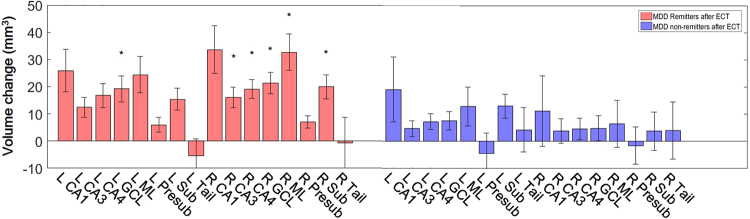
The volume increase of hippocampal subfields in remitters and non-remitters of ECT. The asterisks show significant increases in left GCL and right CA3, CA4, GCL, ML and Sub in remitters of ECT with the Bonferroni correction. MDD, major depressive disorder; CA, cornu ammonis; GCL, granule cell layer; ML, molecular layer; Presub, presubiculum; Sub, subiculum and Tail, hippocampal tail. L, left; R, right.

We found significant effect of ECT on hippocampal subfield volumes with repeated-measurement ANOVA (p = 0.001). In the post-hoc pair t-tests, we found significant volume increases in CA1, CA3, CA4, GCL, ML and Sub in both sides of hippocampus, as well as whole volume increases of both left and right hippocampus (all uncorrected p-values < 0.05), although only left CA3, CA4, GCL, Sub, and right CA4 and GCL were significant after the Bonferroni correction. Post-hoc analysis found the significant increases of volumes in bilateral GCL and right CA3, CA4, ML and Sub of the remitters (Fig. 3).

### Results of correlation analyses

We did not find any correlation between the pre-ECT hippocampal subfield and whole volumes and pre-ECT HAM-D scores (all corrected p-values > 0.05). We also did not find any correlation between the changes of hippocampal subfield and whole volumes with the HAM-D scores before or after ECT, or the change of HAM-D scores (all corrected p > 0.05).

We observed negative correlations between the response to ECT (decrease of HAM-D scores after ECT) and baseline volumes of bilateral CA3 (left: r = −0.64, p = 0.001; right: r = −0.60, p = 0.002), and CA4 (left: r = −0.63, p = 0.001; right: r = −0.62, p = 0.001), GCL (left: r = −0.61, p = 0.002; right: r = −0.61, p = 0.001), as well as right ML (r = −0.65, p = 0.001), Presub (r = −0.70, p < 0.001), Sub (r = −0.71, p < 0.001) and right whole hippocampal volume (r = −0.59, p = 0.002). All p-values survived the Bonferroni correction. These results indicated that patients with smaller hippocampal subfields might achieve betters outcome from ECT.

### Results of predicting ECT responses using hippocampal subfield volumes and machine learning

**Figure 4:**
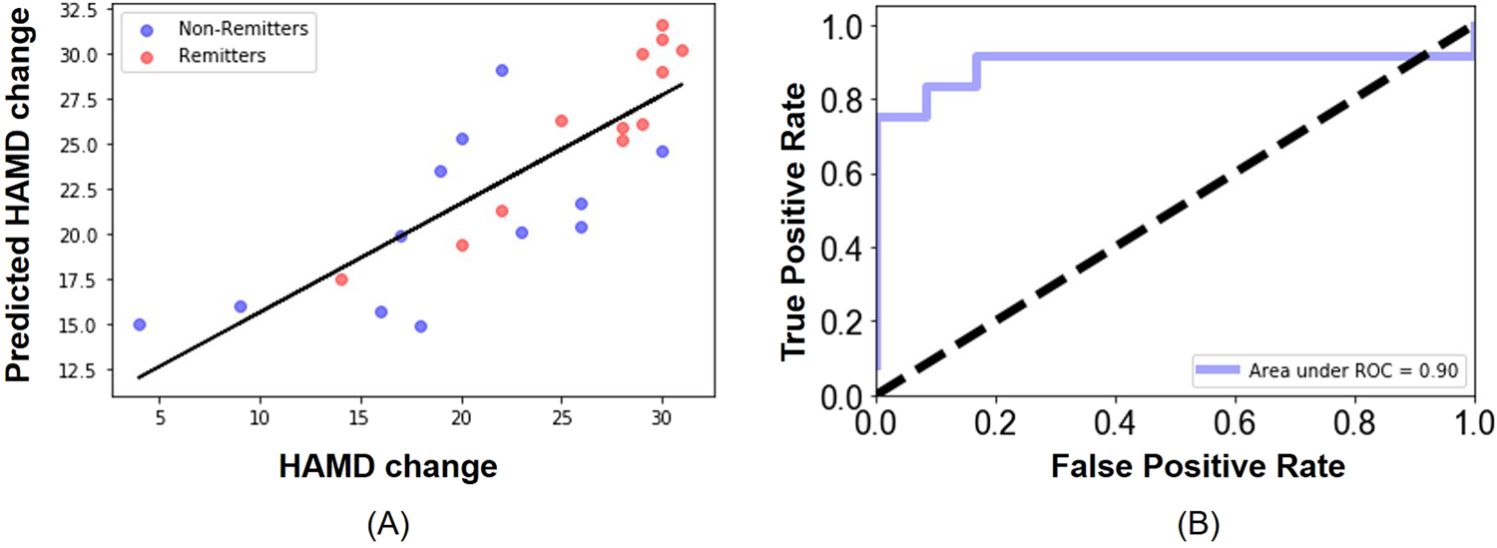
Predicting treatment response to ECT with the volumes of hippocampal subfields and a machine learning algorithm, SVR. (**A**) The prediction of the HAM-D changes that indicates the response to ECT was highly accurate at the individual level (r = 0.81). (**B**) The ROC curve of predicting remitters based on the predicted HAM-D changes. The area under the curve is 0.90.

We could successfully predict the outcome of ECT for each patient by using hippocampal subfield volumes and the machine learning algorithm, which was not possible by using only the whole hippocampal volumes. The correlation coefficient between the actual HAM-D change and the predicted HAM-D change was 0.81 (p < 0.0001; Fig. 4). For remitters alone, the correlation coefficient between the actual HAM-D change and the predicted HAM-D change was as high as 0.93 (p < 0.0001). The volumes of bilateral CA3, Presub, and Sub, as well as the left CA1, ML and right Tail contributed the most to the prediction of the HAM-D change. All except the right Tail showed significantly correlation with the HAM-D change indicating that smaller volumes of these subfields might imply a better treatment response to ECT.

The ROC analysis showed that the predicted depression symptom generated from our model could accurately predict the remitters with an area under the ROC curve (AUC) of 0.90. The sensitivity to predict the remitters was 91.7% and specificity was 75% (overall accuracy 83.3%).

## Discussion

Our findings of ECT-induced volume increases in several hippocampal subfields, such as CA1, CA3, CA4, ML and Sub, especially bilateral GCL and right CA3, CA4, ML and Sub in the remitters, are consistent with previous studies that found volume increases of the gray matter and shape changes^6,11,12,21^, but provided more specific information of the locations affected by ECT than these prior studies. With the help of a machine learning algorithm, the hippocampal subfield volumes at baseline could also make it possible for us to accurately predict whether a patient could achieve remission after ECT and the degree of alleviation of depressive symptoms by ECT for each patient. The performance of the algorithm was comparable to recent studies using machine learning with whole brain gray matter and connectivity as predictors^5,6^. Along with these efforts on individual prediction of ECT outcome, our findings show that focused strategies involving hippocampal subfields using machine learning may help psychiatrists and patients in a more personalized clinical decision making process regarding ECT for treating MDD.

The result of increased volumes of the GCL in the dentate gyrus is consistent with the neurogenesis hypothesis of ECT effect. The GCL is associated with functional neurogenesis during brain development and adulthood^24^. Reduced neurogenesis is linked with stress and mood disorders^25^, and can be recovered by certain interventions, such as antidepressant treatments^26–28^. The response to the antidepressant treatments might be disrupted without neurogenesis in GCL^29^. Animal studies showed that electroconvulsive shocks could induce increased neurogenesis of granule cells in the dentate gyrus^30,31^. Our study provides further imaging evidence *in vivo* in human brain that the layer of granule cells increased in volume after ECT.

The volume increase of other subfields, such as CA3, CA4, ML and subiculum may support the possible synaptogenesis induced by ECT, as these subfields involve intensive synaptic connections to the cortex and to other subfields within the hippocampus and are affected by mood disorders^15,32,33^. The synaptogenesis might be partly due to the neurogenesis in the dentate gyrus that lead to new synaptic connections from the newborn neurons, as well as due to local synaptic remodeling in these subfields, especially in CA3^34,35^. These findings are in line with increased functional connectivity of hippocampus after ECT as observed in other studies^21,36^.

The finding that we could predict responses to ECT in MDD using hippocampal subfield volumes confirmed the rich information provided by these volumes. The prediction was not possible if we used only the whole hippocampal volumes, or if the hippocampal segmentation was not reliable. One study reported that smaller whole hippocampal volume might be related to better outcome of ECT^11^. Our results showed that lower volumes of specific subfields, such as CA3, CA4, GCL, ML and subiculum were associated with better outcomes of ECT. Further investigations on the relationship between the hippocampal subfield volumes and neuro- or synapto-genesis or other biological markers will be necessary to explain why smaller subfield volumes were associate with better outcomes of ECT^37^ (see also an amygdala study^38^). Nevertheless, it was surprising though that by using hippocampal subfield volumes alone we could reach an accuracy of individual predictions that were comparable to studies using the whole brain gray matter and functional connectivity^5,6^. The high accuracy could possibly be attributed to the new, refined segmentation method of hippocampal subfields, which might provide better subfield estimation^14,15,21–23^.

Several limitations should be taken into account when the findings of our study were interpreted. Although our sample size was comparable to several studies in the literature, the findings would be buttressed by replication in larger sample studies. On the other hand, the Bonferroni correction we used in the statistical analysis might over-correct the results, which could be overcome by other correction methods when the sample size is large enough^39,40^. We also did not have controls without MDD who went through interventions comparable to ECT. Even though the cross-validation and SVR algorithm might help to prevent overfitting and provide generalization of methods and findings, further validation on larger and independent samples, preferably from multiple centers^41^, such as the Global ECT-MRI Research Collaboration (GEMRIC)^42^, will be necessary. The sample size of the current study also limited the possibility to fully explore the best features and algorithm with sufficient validations and to optimize for different populations (e.g., sex, stage) over lifespan^43–48^. Although the cross-validation procedure confirmed that the novel segmentation method of hippocampal subfields was reliable, further validation of the anatomical accuracy using i*n vitro* brain tissues and manual tracing on a large sample might still be necessary. Due to the referral system during the recruitment of the inpatients, we could not fully control for any medication effects from prior treatment, and we did not have a third MRI scan that was at least six months after ECT sessions ended compared to some prior studies, although we provided insights regarding the internal regions within the hippocampus that these studies hoped to address^10,12^.

## Conclusion

In the current study, we found that ECT induced volume increase in CA2/3, CA4, GCL, ML and subiculum using a state-of-the-art hippocampal segmentation approach. We accurately predicted the quantitative efficacy of ECT for each patient and whether a patient could achieve remission after ECT by using machine learning and hippocampal subfield volumes at baseline. Our findings provide refined anatomic specificity within the hippocampus as the basis of treatment response to ECT, which may lead to the development of novel pharmacological and neurostimulation treatments, as well as focused targets for future investigations of the cellular and molecular mechanisms of ECT. We also provide a practical approach for informing personalized clinical decision making regarding ECT as a treatment for MDD and predictors of expected clinical outcome employing neuroimaging measures of hippocampal subfields and machine learning approaches. This approach may be generalized to predictions of ECT outcomes in other neurological disorders.

## Materials and Methods

### Participants

Patients with MDD were recruited from inpatient units at the Mental Health Center, the First Affiliated Hospital of Chongqing Medical University. Healthy subjects were recruited through the local community as controls (HC). All patients met Diagnostic and Statistical Manual (DSM)-IV criteria for MDD^49^ and were in a unipolar depressive episode. Diagnoses and structured clinical interviews for DSM-IV were performed by three professional psychiatrists (Q.L., Y.F. and T.Q.). The 24-item Hamilton Depression (HAM-D) Rating Scale was used to assess severity of the symptoms^50,51^, and was evaluated by three professional psychiatrists (Q.L., Y.F. and T.Q.). A total HAM-D score of equal or less than 7 was considered as remission. All the patients were under severe depression and were actively seeking effective treatment. ECT was referred by each patient’s psychiatrist. Every patient underwent a physical examination, a blood test, electroencephalogram, electrocardiogram, and an X-ray exam before ECT. The subjects had not received antipsychotics, antidepressants, mood stabilizers for at least one month. Additional inclusion criteria for the patients included: 1) agreement to take ECT from both the patients and their direct relatives; 2) age from 16 to 60; 3) HAM-D total scores greater or equal to 21 and showing severe symptoms, such as stupor, refusal to take food, self-harming or suicidal behaviors; 4) having no previous or contraindication to ECT treatments. Exclusion criteria for all subjects were: 1) contraindication to MRI scanning; 2) neurological disorders; 3) severe somatic disease; 4) substance abuse; 5) pregnancy; 6) lactation; or 7) depression caused by or combined with somatic disease and other psychiatric disorders. HC must have no self or family history of any psychiatric disorder. All patients and controls were Han Chinese and right-handed. Other demographic information including gender, age and educational level was also collected. This study was approved by the local Institutional Review Board of the Chongqing Medical University in agreement with the Declaration of Helsinki. All research was performed in accordance with relevant guidelines and regulations. All patients and HC signed the written informed consent before participating the study.

### Modified Electroconvulsive Therapy

All patients received eight sessions of modified ECT^52^ within a three-week period: three times per week (Monday, Wednesday, and Friday mornings) for two weeks, and another two times (Monday and Friday mornings) for the third week. The patients were restricted from water and food intake from the midnight before ECT. The patients received MRI scanning and HAM-D rating on the day before the first ECT (pre-ECT or baseline) and the day after the eighth ECT (post-ECT). During the three-week period of ECT, patients did not use any antidepressants or antipsychotics.

ECT was conducted using a Thymatron DGx (Somatics LLC, Lake Bluff, IL) at the Mental Health Center, the First Affiliated Hospital of Chongqing Medical University. The d’Elia placement was used for the standard bitemporal placement of electrodes or bilateral ECT. The initial dosage was selected based on sex, age, weight, and height, and the stimulus intensity was individually adjusted by the seizure response and adverse effects during ECT. Seizure threshold was measured at the first ECT session, which was defined as the smallest electrical dose of producing a seizure of at least 25 seconds on the electroencephalogram^53^. The electrical dosage was set at 1.5–2 times seizure threshold in consecutive ECT sessions according to the extent of seizure^54^. Anesthesia was induced with intravenous atropine (0.5 mg), propofol (1.5–3 mg/kg) and succinylcholine (0.8–2.0 mg/kg). Vital signs were monitored and continuous oxygen inhalation was maintained.

### MRI data acquisition and preprocessing

All MRI scans were performed using a 3 T MRI scanner (Sigma, GE Medical Systems, Waukesha, WI) using a circular polarized birdcage head coil. 3-D T1-weighted images were acquired sagittally using the spoiled gradient recall (SPGR) sequence with the following parameters: echo time = 3.27 ms; repetition time = 8.35 ms; flip angle = 12°; field of view = 240 mm; image matrix = 512 × 512; slice thickness = 1 mm; voxel size = 0.47 × 0.47 × 1 mm^3^; number of slices = 156. The total acquisition time was about 7 min. The subjects were instructed to keep still and no apparent head motion was detected during the scan. The HC were only scanned once at the baseline.

Subcortical reconstruction and segmentation were conducted using the FreeSurfer software (version 5.3.0; http://surfer.nmr.mgh.harvard.edu). The procedure included intensity normalization, automated topology corrections and automatic segmentations of cortical and subcortical regions, which is documented elsewhere^55–57^.

A novel automated algorithm that was included in FreeSurfer was used to segment the hippocampal subfields. The hippocampal subfield atlas was derived from high resolution (0.13 mm) *ex vivo* MRI data of postmortem medial temporal tissue from a 7-T scanner (Fig. 1)^22^. The algorithm was demonstrated to be more accurate than the previous method^58^ and was able to identify granule cell layer (GCL) within the dentate gyrus, the molecular layer (ML) within the subiculum and the CA subfields, as well as the hippocampal tail (Tail; the posterior end of the hippocampus). The algorithm could also provide a better estimation of CA subfield volumes^14^. We included eight hippocampal subfields in the current study: CA1, CA2 and CA3 (noted as CA3 due to the indistinguishable MR contrast between CA2 and CA3), CA4, GCL, ML, presubiculum (Presub), subiculum (Sub) and the Tail.

We used a two-step quality control protocol, similar to the ENIGMA protocol (http://enigma.ini.usc.edu/)^15,59–61^. Each segmented image, overlaid on the corresponding brain structural image, was visually inspected by one of the authors (BC), in order to exclude segmentations with poor registration to the hippocampus location or with apparent wrong assignment of the subfields. Any apparent outlier (five standard deviations) of any hippocampal subfield volume was also excluded. A strict five standard deviation threshold was used to directly exclude any subject due to the substantial individual differences in the hippocampal subfields. We did not exclude any image, because we did not find bad segmentation of hippocampal subfields with the novel algorithm or find any apparent outlier of the subfield volumes.

### Statistical Analyses

Statistical analyses were performed using SPSS (Version 24.0; IBM Corp., Armonk, NY). The efficacy of ECT was evaluated with the average reduction of HAM-D scores, as well as the proportions of patients who had more than 50% decrease of HAM-D scores and whose HAM-D scores became equal or less than 7 after ECT. The patients with HAM-D scores equal or less than 7 after ECT were considered as remitters. For each hippocampal subfield, we used a general linear model (GLM) to investigate the effect of diagnosis. For the MDD group, only the hippocampal subfield volumes before ECT were used. Diagnosis group (HC and MDD) was the independent variable, while the whole hippocampal volume and the hippocampal subfield volumes were the dependent variables. We used age, gender, education and the intracranial volume (ICV) as covariates. Post-hoc analysis was also performed between HC, MDD remitters and MDD non-remitters for each hippocampal subfield. The Bonferroni correction was used for the 18 comparisons (16 subfields plus two whole hippocampal volumes).

To investigate the effect of ECT in patients with MDD, we performed a repeated-measurement ANOVA of all hippocampal subfield volumes before and after ECT (ECT treatment and subfields were all within-subject variables). A post-hoc paired t-test for each hippocampal subfield using the pre-ECT and post-ECT volumes of all MDD patients, as well as remitters and non-remitters. The Bonferroni correction was used for the 18 comparisons for each analysis.

In order to investigate the relationship between the hippocampal subfields and the depressive severity at baseline, we performed correlation analyses between the pre-ECT hippocampal subfield volumes and pre-ECT HAM-D scores, as well as the change of HAM-D scores (pre-ECT minus post-ECT). We also performed correlation analyses between the change of hippocampal subfield volumes and the change of HAM-D scores to investigate the relationship between the hippocampal subfield changes and the depressive severity changes due to ECT. We considered p-values < 0.0028 (0.05/18) significant, and the raw p-values were reported.

### Predicting ECT responses using hippocampal subfield volumes and machine learning

The major focus of the present study was to investigate whether hippocampal subfield volumes at baseline could predict the future outcome of ECT at an individual level. The outcome of ECT could be measured by the change of HAM-D total scores. A positive outcome should be indicated as a significant decrease of the HAM-D total score from the HAM-D score at baseline.

We used the linear kernel support vector regression (SVR) to predict the HAM-D change of each patient using the hippocampal subfield volumes. The volume of each subfield was normalized to the normal distribution (individual volumes subtracted the mean and then divided by the standard deviation of each subfield of all the subjects) before being used as the input feature to SVR. We used the default settings in the “sklearn” package of python. Leave-one-out cross-validation (LOOCV) was used, where one subject was left out iteratively as the testing target and the rest of the sample was used to train the SVR. Within the training during cross-validation, a linear SVR was used to estimate the weight of the features for the patient identification model and the features were ranked based on their weights. An internal LOOCV was used to determine the number of features that should be used for the predicting model by calculating the accuracy of the top N features and selecting the N that generated the best accuracy (N ranged from 1 to 16). The resultant model was then used to predict the HAM-D change of the testing patient. The performance of the algorithm was evaluated using the Pearson’s correlation of the actual HAM-D changes and the predicted HAM-D changes.

Based on the predicted HAM-D changes and the actual category of remission, we calculated the accuracy of predicted remitters and performed a receiver operating characteristic (ROC) analysis to evaluate the accuracy of our predictions with respect to remission.

## References

[1] Whiteford HA (2013). Global burden of disease attributable to mental and substance use disorders: Findings from the Global Burden of Disease Study 2010. Lancet.

[2] Murray CJL (2012). Disability-adjusted life years (DALYs) for 291 diseases and injuries in 21 regions, 1990-2010: a systematic analysis for the Global Burden of Disease Study 2010. Lancet.

[3] Lisanby SH (2007). Electroconvulsive Therapy for Depression. N Engl J Med.

[4] Janicak, P. G. *et al*. Efficacy of ECT: A Meta-Analysis. *Am. J. Psychiatry* 297–302 (1985). 10.1176/ajp.142.3.297 3882006

[5] Redlich R (2016). Prediction of Individual Response to Electroconvulsive Therapy via Machine Learning on Structural Magnetic Resonance Imaging Data. JAMA psychiatry.

[6] van Waarde JA (2015). A functional MRI marker may predict the outcome of electroconvulsive therapy in severe and treatment-resistant depression. Mol. Psychiatry.

[7] Wennström M, Hellsten J, Tingström A (2004). Electroconvulsive seizures induce proliferation of NG2-expressing glial cells in adult rat amygdala. Biol. Psychiatry.

[8] Hellsten J (2002). Electroconvulsive seizures increase hippocampal neurogenesis after chronic corticosterone treatment. Eur. J. Neurosci..

[9] Chen F, Madsen TM, Wegener G, Nyengaard JR (2009). Repeated electroconvulsive seizures increase the total number of synapses in adult male rat hippocampus. Eur. Neuropsychopharmacol..

[10] Dukart J (2014). Electroconvulsive therapy-induced brain plasticity determines therapeutic outcome in mood disorders. Proc. Natl. Acad. Sci. USA.

[11] Joshi SH (2016). Structural plasticity of the hippocampus and amygdala induced by electroconvulsive therapy in major depression. Biol. Psychiatry.

[12] Nordanskog P, Larsson MR, Larsson EM, Johanson A (2014). Hippocampal volume in relation to clinical and cognitive outcome after electroconvulsive therapy in depression. Acta Psychiatr. Scand..

[13] Small SA, Schobel SA, Buxton RB, Witter MP, Barnes CA (2011). A pathophysiological framework of hippocampal dysfunction in ageing and disease. Nat. Rev. Neurosci..

[14] Ho, N. F. *et al*. Progression from selective to general involvement of hippocampal subfields in schizophrenia. *Mol. Psychiatry* 1–11 10.1038/mp.2016.4 (2016). 10.1038/mp.2016.4 PMC4995163 26903271

[15] Cao, B. *et al*. Hippocampal subfield volumes in mood disorders. *Mol. Psychiatry*, 10.1038/mp.2016.262 (2017). 10.1038/mp.2016.262 PMC5524625 28115740

[16] Baglivo, V. *et al*. Hippocampal Subfield Volumes in Patients With First-Episode Psychosis Valentina. *FEMS Microbiol. Ecol*. 1–8 (2017). 10.1093/femsec/fix097 10.1093/schbul/sbx108 PMC5890476 29897598

[17] Ho NF (2017). Progressive Decline in Hippocampal CA1 Volume in Individuals at Ultra-High-Risk for Psychosis Who Do Not Remit: Findings from the Longitudinal Youth at Risk Study. Neuropsychopharmacology.

[18] Cao B (2016). Hippocampal volume and verbal memory performance in late-stage bipolar disorder. J. Psychiatr. Res..

[19] Zeni, C. P. *et al*. Interaction between BDNF rs6265 Met allele and low family cohesion is associated with smaller left hippocampal volume in pediatric bipolar disorder. *Journal of Affective Disorders* **189**, 94–97 (2016). 10.1016/j.jad.2015.09.031 PMC4733573 26432032

[20] Cao, B. *et al*. Reduced hippocampus volume and memory performance in bipolar disorder patients carrying the BDNF val66met met allele. *Journal of Affective Disorders* **198**, 198–205 (2016). 10.1016/j.jad.2016.03.044 PMC5214589 27018938

[21] Abbott CC (2014). Hippocampal structural and functional changes associated with electroconvulsive therapy response. Transl. Psychiatry.

[22] Iglesias JE (2015). A computational atlas of the hippocampal formation using *ex vivo*, ultra-high resolution MRI: Application to adaptive segmentation of *in vivo* MRI. Neuroimage.

[23] Haukvik UK (2015). *In vivo* hippocampal subfield volumes in schizophrenia and bipolar disorder. Biol. Psychiatry.

[24] van Praag H (2002). Functional neurogenesis in the adult hippocampus. Nature.

[25] Gould E, Tanapat P, McEwen BS, Flügge G, Fuchs E (1998). Proliferation of granule cell precursors in the dentate gyrus of adult monkeys is diminished by stress. Proc. Natl. Acad. Sci. USA.

[26] Drew MR, Hen R (2007). Adult hippocampal neurogenesis as target for the treatment of depression. CNS Neurol. Disord. Drug Targets.

[27] Malberg JE, Eisch aJ, Nestler EJ, Duman RS (2000). Chronic antidepressant treatment increases neurogenesis in adult rat hippocampus. J. Neurosci..

[28] Warner-Schmidt JL, Duman RS (2006). Hippocampal neurogenesis: Opposing effects of stress and antidepressant treatment. Hippocampus.

[29] Santarelli L (2003). Requirement of Hippocampal Neurogenesis for the Behavioral Effects of Antidepressants. Science (80-)..

[30] Scott BW, Wojtowicz JM, Burnham WM (2000). Neurogenesis in the Dentate Gyrus of the Rat Following Electroconvulsive Shock Seizures. Exp. Neurol..

[31] Madsen, T. M. *et al*. Increased Neurogenesis in a Model of Electroconvulsive Therapy n d u c t o i n o f s e z i u r e s n t h e f o r m o f e e l c t r o c o n v u s l v i e. 3223, 50006 (2000). 10.1016/s0006-3223(00)00228-6 10862803

[32] O’Mara S (2005). The subiculum: What it does, what it might do, and what neuroanatomy has yet to tell us. J. Anat..

[33] Freund TF, Buzsáki G (1996). Interneurons of the hippocampus. Hippocampus.

[34] Markakis EA, Gage FH (1999). Adult-generated neurons in the dentate gyrus send axonal projections to field CA3 and are surrounded by synaptic vesicles. J. Comp. Neurol..

[35] Hajszan T, MacLusky NJ, Leranth C (2005). Short-term treatment with the antidepressant fluoxetine triggers pyramidal dendritic spine synapse formation in rat hippocampus. Eur. J. Neurosci..

[36] Qiu H (2016). Electroconvulsive Therapy-Induced Brain Structural and Functional Changes in Major Depressive Disorders: A Longitudinal Study. Med. Sci. Monit..

[37] Dean J, Keshavan M (2017). The neurobiology of depression: An integrated view. Asian J. Psychiatr..

[38] Redlich R (2017). Effects of electroconvulsive therapy on amygdala function in major depression - A longitudinal functional magnetic resonance imaging study. Psychol. Med..

[39] Sankoh AJ, D’Agostino RB, Huque MF (2003). Efficacy endpoint selection and multiplicity adjustment methods in clinical trials with inherent multiple endpoint issues. Stat. Med..

[40] Pocock SJ (2001). A score for predicting risk of death from cardiovascular disease in adults with raised blood pressure, based on individual patient data from randomised controlled trials. BMJ.

[41] Redlich R (2014). Brain morphometric biomarkers distinguishing unipolar and bipolar depression: A voxel-based morphometry-pattern classification approach. JAMA Psychiatry.

[42] Oltedal L (2017). The Global ECT-MRI Research Collaboration (GEMRIC): Establishing a multi-site investigation of the neural mechanisms underlying response to electroconvulsive therapy. NeuroImage Clin..

[43] Cao B (2015). Development and validation of a brain maturation index using longitudinal neuroanatomical scans. Neuroimage.

[44] Cao, B. *et al*. Lifespan Gyrification Trajectory of Human Brain and Accelerated Aging of Major Psychiatric Disorders. (2016).

[45] Lavagnino L (2015). Changes in the corpus callosum in women with late-stage bipolar disorder. Acta Psychiatr. Scand..

[46] Mwangi B (2016). Individualized Prediction and Clinical Staging of Bipolar Disorders Using Neuroanatomical Biomarkers. Biol. Psychiatry Cogn. Neurosci. Neuroimaging.

[47] Cao, B. *et al*. Brain gyrification and neuroprogression in bipolar disorder. *Acta Psychiatr. Scand*. **135** (2017). 10.1111/acps.12738 PMC7083164 28430365

[48] Hasan, K. M. *et al*. Entorhinal Cortex Thickness across the Human Lifespan. *J. Neuroimaging* **26** (2016). 10.1111/jon.12297 PMC4826319 26565394

[49] American Psychiatric Association. Diagnostic and statistical manual of mental disorders (4th ed.). Diagnostic and statistical manual of mental disorders (4th ed.) (1994).

[50] Hamilton (1960). M. C. Hamilton Depression Rating Scale (HAM-D). Redloc.

[51] Hamilton M (1980). Rating depressive patients. J. Clin. Psychiatry.

[52] Rudorfer, M., Henry, M. & Sackeim, H. In Psychiatry, Second Edition. (eds Tasman, A., Kay, J. & Lieberman, J.) (Chichester: John Wiley & Sons Ltd, 2003).

[53] Abrams, R. Electroconvulsive therapy (4th ed.). Electroconvulsive therapy (4th ed.). (2002).

[54] Kennedy, S. H., Lam, R. W., Parikh, S. V., Patten, S. B. & Ravindran, A. V. Canadian Network for Mood and Anxiety Treatments (CANMAT) Clinical guidelines for the management of major depressive disorder in adults. *J. Affect. Disord*. **117** (2009). 10.1016/j.jad.2009.06.043 19682750

[55] Dale AM, Fischl B, Sereno MI (1999). Cortical surface-based analysis. I. Segmentation and surface reconstruction. Neuroimage.

[56] Jovicich J (2006). Reliability in multi-site structural MRI studies: Effects of gradient non-linearity correction on phantom and human data. Neuroimage.

[57] Fischl B (2002). Whole brain segmentation: Automated labeling of neuroanatomical structures in the human brain. Neuron.

[58] Van Leemput K (2009). Automated segmentation of hippocampal subfields from ultra-high resolution *in vivo* MRI. Hippocampus.

[59] Schmaal, L. *et al*. Cortical abnormalities in adults and adolescents with major depression based on brain scans from 20 cohorts worldwide in the ENIGMA Major Depressive Disorder Working Group. *Mol. Psychiatry*, 10.1038/mp.2016.60 (2016). 10.1038/mp.2016.60 PMC5444023 27137745

[60] Hibar, D. P. *et al*. Subcortical volumetric abnormalities in bipolar disorder. *Mol. Psychiatry*, 10.1038/mp.2015.227 (2016). 10.1038/mp.2015.227 PMC5116479 26857596

[61] Schmaal, L. *et al*. Subcortical brsain alterations in major depressive disorder: findings from the ENIGMA Major Depressive Disorder working group. *Mol. Psychiatry* 1–7 10.1038/mp.2015.69 (2015). 10.1038/mp.2015.69 PMC4879183 26122586

