# Author Correction: Predicting individual responses to the electroconvulsive therapy with hippocampal subfield volumes in major depression disorder

**DOI:** 10.1038/s41598-020-73418-0

**Published:** 2020-10-12

**Authors:** Bo Cao, Qinghua Luo, Yixiao Fu, Lian Du, Tian Qiu, Xiangying Yang, Xiaolu Chen, Qibin Chen, Jair C. Soares, Raymond Y. Cho, Xiang Yang Zhang, Haitang Qiu

**Affiliations:** 1 Mental Health Center, The First Affiliated Hospital of Chongqing Medical University, Chongqing, People’s Republic of China; 2 Department of Psychiatry and Behavioral Sciences, McGovern Medical School, The University of Texas Health Science Center at Houston, Houston, USA; 3 Department of Anesthesiology, The First Affiliated Hospital of Chongqing Medical University, Chongqing, People’s Republic of China

Correction to:* Scientific Reports* 10.1038/s41598-020-73418-0, published online 3 April 2018

In the original version of this Article, Bo Cao was incorrectly affiliated with ‘Mental Health Center, The First Affiliated Hospital of Chongqing Medical University, Chongqing, P. R. China’. The correct affiliation is listed below.

Department of Psychiatry and Behavioral Sciences, McGovern Medical School, The University of Texas Health Science Center at Houston, Houston, United States.

In addition, the Results section contained a typographical error. Under the subheading ‘Demographics’ the sentence,

“Patients with MDD had an average of HAM-D total scores as high as 31.3, which was significantly different from that of HC (F_1,31_ = 544.505, p < 0.0001; one HC had missing HAM-D scores).”

now reads:

“Patients with MDD had an average of HAM-D total scores as high as 31.3, which was significantly different from that of HC (F_1,36_ = 544.505, p < 0.0001; one HC had missing HAM-D scores).”

These errors have now been corrected in the PDF and HTML versions of the Article.

